# The NLR SkinApp: Testing a Supporting mHealth Tool for Frontline Health Workers Performing Skin Screening in Ethiopia and Tanzania

**DOI:** 10.3390/tropicalmed9010018

**Published:** 2024-01-10

**Authors:** Nelly Mwageni, Robin van Wijk, Fufa Daba, Ephrem Mamo, Kitesa Debelo, Benita Jansen, Anne Schoenmakers, Colette L. M. van Hees, Christa Kasang, Liesbeth Mieras, Stephen E. Mshana

**Affiliations:** 1 Department of Microbiology, Catholic University of Health and Allied Sciences, Mwanza 33102, Tanzania; nellymwageni@yahoo.com (N.M.);; 2 NLR/Leprastichting, 1097 DN Amsterdam, The Netherlandsa.schoenmakers@nlrinternational.org (A.S.); l.mieras@nlrinternational.org (L.M.); 3 Department of Public Health, Erasmus University Medical Center, 3015 GD Rotterdam, The Netherlands; 4 Deutsche Lepra-und Tuberkulosehilfe, Addis Ababa 1165, Ethiopia; fufa.daba@dahw.org (F.D.); kitesa.debelo@dahw.org (K.D.); 5 Armauer Hansen Research Institute, Addis Ababa 1005, Ethiopia; ephremmamo2015@gmail.com; 6 Aklilu Lemma Institute of Pathobiology, Addis Ababa University, Addis Ababa 1176, Ethiopia; 7 Department of Dermatology, Erasmus University Medical Center, 3015 GD Rotterdam, The Netherlands; c.vanhees@erasmusmc.nl; 8 Deutsche Lepra-und Tuberkulosehilfe, 97080 Würzburg, Germany; dr.christa.kasang@dahw.de

**Keywords:** mHealth, eHealth, health app, dermatology, neglected tropical diseases, leprosy, Ethiopia, Tanzania

## Abstract

**Background:** The prevalence of skin diseases such as leprosy, and limited dermatological knowledge among frontline health workers (FHWs) in rural areas of Sub-Saharan Africa, led to the development of the NLR SkinApp: a mobile application (app) that supports FHWs to promptly diagnose and treat, or suspect and refer patients with skin diseases. The app includes common skin diseases, neglected tropical skin diseases (skin NTDs) such as leprosy, and HIV/AIDS-related skin conditions. This study aimed to test the supporting role of the NLR SkinApp by examining the diagnostic accuracy of its third edition. **Methods:** A cross-sectional study was conducted in East Hararghe, Ethiopia, as well as the Mwanza and Morogoro region, Tanzania, in 2018–2019. Diagnostic accuracy was measured against a diagnosis confirmed by two dermatologists/dermatological medical experts (reference standard) in terms of sensitivity, specificity, positive predictive value, and negative predictive value. The potential negative effect of an incorrect management recommendation was expressed on a scale of one to four. **Results:** A total of 443 patients with suspected skin conditions were included. The FHWs using the NLR SkinApp diagnosed 45% of the patients accurately. The values of the sensitivity of the FHWs using the NLR SkinApp in determining the correct diagnosis ranged from 23% for HIV/AIDS-related skin conditions to 76.9% for eczema, and the specificity from 69.6% for eczema to 99.3% for tinea capitis/corporis. The inter-rater reliability among the FHWs for the diagnoses made, expressed as the percent agreement, was 58% compared to 96% among the dermatologists. Of the management recommendations given on the basis of incorrect diagnoses, around one-third could have a potential negative effect. **Conclusions:** The results for diagnosing eczema are encouraging, demonstrating the potential contribution of the NLR SkinApp to dermatological and leprosy care by FHWs. Further studies with a bigger sample size and comparing FHWs with and without using the NLR SkinApp are needed to obtain a better understanding of the added value of the NLR SkinApp as a mobile health (mHealth) tool in supporting FHWs to diagnose and treat skin diseases.

## 1. Introduction

Skin diseases are considered to be one of the most common human health issues worldwide [1]. With a high prevalence in both low- and high-income countries, they permeate all civilizations [2]. However, skin diseases are more prevalent in low-income countries. Available data of epidemiological studies on skin diseases in low-income countries have found an overall point prevalence in some areas up to 62.2% [3,4,5,6].

The Global Burden of Disease 2010 study investigated the global burden caused by skin diseases. It was found to be the fourth leading cause of nonfatal burden in the world [1]. Although skin diseases are in most cases nonfatal, the influence they can have on the quality of life of the affected individual is often significant, especially in low-income countries, where skin diseases are undertreated due to little or no access to dermatological health care services [4,7].

Sub-Saharan Africa is home to five countries, where nearly half of the impoverished population of this region reside, including Tanzania and Ethiopia [8]. The population of these countries is unevenly distributed, with the majority of people living in rural areas. In 2019, the rural population of Tanzania and Ethiopia accounted for 66% and 79%, respectively, with a significant discrepancy in rural–urban disparity in terms of health care coverage caused by long travel distances, poor quality of basic healthcare services, and a shortage of doctors in rural areas [9,10,11,12]. The shortage of doctors in Sub-Saharan Africa, particularly medical specialists, remains a critical issue. Ethiopia counts 0.77 doctors per 10,000 inhabitants; in Tanzania this is 0.14 [12,13]. In addition, no dermatologists are available in most areas in both countries [14]. Currently, the majority of the rural population with skin diseases is served by frontline health workers (FHWs) with limited dermatological knowledge [15,16]. These health workers will not only face the more common skin diseases, such as eczema, scabies, tinea capitis, psoriasis, and acne [1,5,13,17,18], but are also confronted with HIV/AIDs-related skin conditions, as well as skin-related neglected tropical diseases (NTDs). These NTDs, as the name implies, are a group of often overlooked diseases, and most are relatively rare as compared to the common skin diseases [19]. They commonly afflict the world’s poorest populations, with approximately 500 million affected people living in Sub-Saharan Africa [20]. Some NTDs are associated with skin conditions which can lead to disability —such as leprosy and lymphatic filariasis (LF)—, stigmatization, exclusion, and mental health problems [21]. However, because of the low prevalence and the lack of dermatological knowledge, these skin-related NTDs are difficult to recognize by FHWs [16,20].

To bridge the gap between the high prevalence of skin diseases and the lack of knowledge and skills of the available health workers in low-income countries, telemedicine, in particular teledermatology, can be used [22]. Telemedicine is created to deliver medical care by using electronic information and telecommunication technologies, which is especially relevant for patients living in remote areas [23]. Application of this technique in the field of dermatology has demonstrated to be a suitable manner for providing dermatological care, especially in low-resource settings [24]. A patient can be seen directly in real-time through a videoconference, or later be (re-)assessed by exchanging images with a dermatologist. However, there are some limitations concerning teledermatology, for instance the need for a dermatologist to be available as well as the need to have electricity available and a stable internet connection, which can be obstacles in rural areas. Therefore, the use of wireless, offline devices (mobile phones, tablets) to support the healthcare system, also known as mobile health (mHealth), can offer FHWs the opportunity to facilitate dermatological diagnosis and management [16]. mHealth encompasses innovative techniques that enable remote monitoring, improve primary prevention and healthcare outcomes, facilitate self-management of chronic conditions, and reduce the need for patients to visit healthcare facilities, without the direct involvement of a medical specialist [25]. Furthermore, available studies show the beneficial role of mHealth in enhancing the FHWs’ disease knowledge and work performance in low- and middle-income countries [26,27].

In 2015, NLR—a nongovernmental organization (NGO) working in the field of leprosy—used the experience of a successful pilot study with a paper-based decision tree in Nigeria, based on the algorithm of Mahé et al. [28], to develop a mobile application (app) which eventually led to the third version of the NLR SkinApp, an offline functioning mHealth tool [16]. This third version of the application contains 29 skin diseases, divided in three categories: ‘common skin diseases’, ‘skin-related NTDs’, and ‘HIV/AIDS-related skin diseases’ (Table 1).

However, before launching new and further developed versions of the NLR SkinApp and promoting it on larger scale, the validity of diagnoses made with the application has to be determined. Therefore, an NLR SkinApp validation study was performed. The aim of this study was to test the supporting role of the NLR SkinApp by investigating the diagnostic accuracy and reproducibility of its third version, while utilized by FHWs in Ethiopia and Tanzania. Furthermore, the potential negative effects of a management recommendation given by FHWs using the NLR SkinApp in case of an incorrect diagnosis were evaluated.

## 2. Materials and Methods

A cross-sectional study was conducted at outpatient dermatology clinics and regional hospitals in the cultural context of East Hararghe, Ethiopia, as well as the Mwanza and Morogoro region, Tanzania.

### 2.1. Study Population

Patients (age ≥ 2 years) with symptoms and signs of skin diseases presenting at outpatient clinics in Tanzania and Ethiopia were recruited from September 2018 to October 2019 to participate in this study. They were considered eligible if they had not been diagnosed or seen by a health worker before for that specific skin condition. Patients were excluded in case the patient or the patient’s parents were unable to demonstrate or communicate their signs and symptoms or, in the event of a follow-up, they had disclosed their diagnosis to one of the FHWs prior to their examination with the NLR SkinApp. All patients were asked for consent prior to inclusion.

### 2.2. Data Collection

Patients who were considered eligible for the study were seen successively by two FHWs and two dermatologists on the same day in the same health service unit. First, the patient was seen by the two FHWs, who randomly alternated in being first or second. They formulated a diagnosis and advised treatment or referral while using the NLR SkinApp. To avoid bias, they were blinded for the results of their colleague. Subsequently, the patient was seen by two dermatological experts. In Ethiopia, these were a dermatologist and a general physician with extensive training and experiences in the management of dermatological diseases. In Tanzania, two body-certified dermatologists and one dermatology officer (a doctor with an advanced diploma in dermatology) took part in this study. They also formulated a diagnosis and management recommendation independently, while they were blinded for the results of their colleague and both FHWs. Data were collected by FHWs and dermatologists, following a half-day training by and with guidance from NLR staff in which they learned about the functionalities of the SkinApp and how to document their findings on data collection forms.

### 2.3. Outcome Measures

The primary outcome of this study was the percentage of correct diagnoses made by the FHWs using the NLR SkinApp. The FHWs were not asked to assess the patient without the use of SkinApp. A diagnosis was considered correct if it matched the reference standard. The reference standard in this study was defined by two dermatological experts/dermatologists who independently formulated a diagnosis after the patient was seen by the FHW. In addition to being blinded for their dermatologist colleague’s diagnosis, the dermatological experts were also not informed about the FHW’s diagnosis. In this study, a matching diagnosis made by both dermatological experts who assessed the same patient was considered the reference standard diagnosis. In case there was no literal agreement among the dermatological experts, a third dermatologist from The Netherlands with international experience and an NLR staff member experienced in diagnosing and treating tropical skin diseases assessed if agreement could be reached on the reference standard. In case a ‘reference standard’ could not be defined because of missing data (no diagnosis made) or because no agreement could be reached, participants could not be included. Secondary outcomes were inter-rater reliability and the level of harm caused by incorrect diagnosis and management recommendation as a consequence of using the NLR SkinApp. Additionally, management choices made by the FHWs were classified as ‘correct’ or ‘not correct’ by four medical doctors, including a dermatologist from Tanzania, a dermatologist with international experience from The Netherlands, a primary care physician experienced in diagnosing and treating skin diseases from The Netherlands, and a medical doctor specialized in public health and infectious diseases from The Netherlands. Subsequently, they weighed the potential negative effects in case of an incorrect management recommendation as neutral, potential negative effects, or potential severe effects. The definition of these categories can be found in Table 2. In case of discrepancy, consensus was reached following discussions among the four medical doctors involved.

### 2.4. Statistical Analysis

Quantitative data were collected on paper and entered in an Epi Info™ 7 database, from which the data were exported to Microsoft Excel 2010. Data analysis was performed using SPSS, version 27. Basic features in the study population were described by performing a descriptive analysis. For each individual skin disease, a sample size was calculated. For most diseases or disease groups, 27 cases were needed to reach the lowest level of desired precision (15%), sensitivity (80%), and a confidence interval (95%) [29]; this sample size calculation was performed using EpiCalc 2000.

Diagnostic accuracy of the NLR SkinApp was assessed by calculating the sensitivity, specificity, and predictive values [30,31]. The validity of the NLR SkinApp was determined by the inter-rater variability, calculated by means of percent agreement. Additionally, the percent agreement among the dermatologists was calculated as reference standard.

### 2.5. Ethical Considerations

This study was approved by the CUHAS/BMC Research and Ethical Committee (CREC) in Tanzania and the AHRI/ALERT Ethical Review committee (AAERC) in Ethiopia. Patients were only included when they had signed or thumb printed a (parental) informed consent form after being informed about the study and their rights according to the WHO Process of Obtaining Informed Consent [32].

All patients who took part in this study have been diagnosed and treated following the advice of the dermatologist/dermatological medical expert, in line with national medical guidelines.

## 3. Results

Of the 521 participants who were included in the study, 57 (10.9%) participants were excluded for data analysis because of missing diagnosis of one or both FHWs or dermatologists. Another 21 (4.0%) participants were not eligible for data analysis because no diagnosis as reference standard could be formulated because of an inconsistency in diagnosis made by the dermatologists, resulting in a final total of 443 participants who were included for data analysis.

Out of the 443 participants, 53.7% were female and 45.1% were male. In five cases (1.1%), the sex reported by the FHWs was inconsistent and therefore marked as unknown. The age of the participants ranged from 2 to 85 years, with a mean age of 33 (SD 17.9) years. Of the patients analysed, 13.3% were aged 14 years or below. Most participants were seen in Tanzania (82.8%).

### 3.1. Skin Diseases

A varied spectrum of skin diseases was seen. In total, 478 reference standard diagnoses were made in 443 patients; some participants presented with more than one skin disease. Based on the diagnoses included in the NLR SkinApp, eczema was the leading disease group (106–22.2%), followed by acne (28–5.9%), and the diseases pityriasis versicolor (23–4.8%), tinea capitis (17–3.6%), scabies (13–2.7%), and vitiligo (13–2.7%), though most of the skin diseases (202) were classified as ‘other’ (42.3%). In this group, urticaria was the most named skin disease according to the reference standard (32–15.8%), followed by keloid (14–6.9%), folliculitis (13–6.4%), lichen planus (12–5.9%), and tinea pedis (11–5.5%). An overview of the 10 most common skin diseases in the category ‘other’ can be found in Table 3. The category HIV/AIDS-related skin diseases accounted for 15.7% (75) of the cases, and the category skin-related NTDs accounted for 5.9% (28) of the cases.

Eczema was the most common skin condition in the research areas in both Tanzania (23.8%) and Ethiopia (13.9%). Commonly seen skin conditions in Tanzania were acne (6.3%) and pityriasis versicolor (5.3%), whereas in Ethiopia, higher rates of vitiligo (8.9%) and angular cheilitis (7.6%) were seen. Leprosy was diagnosed more often in the research area in Ethiopia (6.3%) compared with the research area in Tanzania (1.8%).

### 3.2. Diagnostic Accuracy

Out of the total 886 separate diagnoses formulated by all FHWs combined by using the NLR SkinApp, 394 (44.5%) diagnoses matched the reference standard diagnosis. As stated above, for most diseases, the calculated sample size was at least 27, based on a level of precision (15%), sensitivity (80%), and a confidence interval (95%). For five (categories of) skin diseases included in the NLR SkinApp, the required number of cases was reached: eczema (n = 106), HIV/AIDS-related skin diseases (n = 74), tinea capitis/corporis (n = 28), acne (n = 28), and skin-related NTDs (n = 28). However, the category ‘skin-related NTDs’, including 10 skin diseases (Table 1), consisted mainly of leprosy and scabies cases, 12 and 13, respectively. Sensitivity was highest for eczema (76.9%). Poor sensitivity was found for skin-related NTDs (37.5%), HIV/AIDS-related skin diseases (23%), and tinea capitis/corporis (26.8%). However, specificity in these groups was high, especially for tinea capitis/corporis (95.7%), followed by skin-related NTDs (92.2%). Lower specificity was found for eczema (69.3%). The positive predictive value was low for each group. The negative predictive value appeared to be relatively high for tinea capitis/corporis and skin-related NTDs (Table 4).

### 3.3. Management Recommendations

A total of 886 separate management recommendations were formulated by the participating FHWs by using the NLR SkinApp, resulting in a final total of 884 management recommendations eligible to be evaluated. Management recommendations given by the FHWs was correct in 398 (45.0%) cases, because it was based on the correct diagnosis. Of the management recommendations given, 486 (54.8%) were based on an incorrect diagnosis. However, of those recommendations based on an incorrect diagnosis, 296 (60.9%) of the management recommendations given were considered ‘neutral’, because the advice given was either referral, was partially beneficial, or had no potential negative effects. Of the incorrect management recommendations, 180 (37.0%) could potentially have ‘negative effects’, for example, when medication is prescribed while it is unnecessary (antibiotics or low-class steroids). Of these, 10 (2.1%) could potentially have ‘severe’ effects. Of the ten recommendations defined as potentially ‘severe’, two cases were defined to be serious—one case concerning a patient with erythema nodosum leprosum in which the FHW advised medication to treat scabies, and another case concerning a patient with systemic sclerosis in which the FHW advised no treatment or referral.

### 3.4. Validity

In context of the inter-rater reliability, a lower percent of agreement was found between the diagnoses made by the FHWs (58%) compared with the dermatologists (96%).

## 4. Discussion

This was the first study to investigate the value of the NLR SkinApp when used by FHWs as a supportive tool for diagnosis and management of skin diseases. The results of this study provided insights into the diagnostic accuracy and reproducibility of the third version of the NLR SkinApp, the potential negative effects that incorrect diagnosis suggested by the app can lead to, and the pattern of skin diseases in the research areas in Ethiopia and Tanzania.

The NLR SkinApp is an mHealth app, focusing on decision support. In addition to the NLR SkinApp, there are more decision support apps in the field of dermatology, such as DermaAID and Dermion [33]. However, data concerning the efficacy of these apps is lacking. So far, no recently published scientific literature is only available on decision support apps that focus solely on one specific skin disease, i.e., early detection of melanomas [33,34]. The NLR SkinApp includes a broader spectrum of skin diseases and is focusing on the patient population in sub-Sahara Africa, allowing the FHWs to serve a larger population. This patient population, as well as photos of conditions presenting on patients with black and brown skin colour, is often underrepresented in international dermatological capacity strengthening materials [14,35].

The current study made use of various statistical measurements to assess the performance level of the FHWs when using the NLR SkinApp, as well as the performance level of the NLR SkinApp. Compared to a previous study using a paper-based decision tree as diagnostic test, which was used to develop the NLR SkinApp, the NLR SkinApp appeared less accurate, with 44.5% of the cases correctly diagnosed by the FHWs using the NLR SkinApp, compared to 82% in the study of Taal et al. [36]. Furthermore, Taal et al.’s study using data from Kano State, Nigeria revealed higher sensitivity and PPV for tinea capitis (94.8% and 91.4%, respectively), but found lower sensitivity and PPV for contact eczema (7.1% and 29.7%, respectively) compared with the findings of this study [36]. No data were available concerning the sensitivity and the PPV of the category ‘skin-related NTDs’. These diseases (except for scabies) have a relatively low prevalence [19]. Regarding the numbers involved and data available, it is therefore challenging for new (supporting) diagnostic tools to compute PPVs [37]. In addition, the ‘skin-related NTDs’ group is very diverse (Table 1), but because of the relative scarcity, separate analysis of the diseases will require a much bigger sample size, which is logistically and financially challenging. Nevertheless, disease integration of these conditions, as well as task-shifting, is promoted by the WHO to increase health care coverage; thus, more research and allocated funding in this field is needed [38,39].

To put the results of the diagnostic accuracy in broader perspective, a study conducted in Uganda, assessing the performance level of an artificial intelligence (AI) dermatological algorithm using images of skin diseases, found low overall diagnostic accuracy: the AI app placed the correct diagnosis in the top five differential diagnoses in 21 of the 123 images (17%) [40]. However, similar to this study, better performance levels of the AI app were found when focusing specifically on dermatitis; correct diagnosis was predicted as most likely diagnosis in 80% of the images [40]. Furthermore, a systematic review of Freeman et al. assessing the diagnostic accuracy of algorithm-based smartphone apps focusing on the risk of skin cancer in adults found a sensitivity and specificity of the SkinVision app of 80% and 78%, respectively [41]. These results were in line with the sensitivity (76.5%) and specificity (69.6%) of the skin category ‘eczema’ found in this study.

Possible explanations regarding the discrepancy in sensitivity and PPV of the different skin categories found in the NLR SkinApp compared to the diagnostic paper-based decision tree used in the study of Taal et al. may be the difference in study design, as well as disease prevalence [36,42,43]. First, the study of Taal et al. used an algorithm in support of the diagnostic process, which consisted of a flowchart with diagnostic steps. This flowchart identified seven of the most common skin diseases. The wider spectrum of skin diseases included in the NLR SkinApp compared to the flowchart used in the study of Taal et al. [36] may have led to differences in sensitivity and specificity between the two studies. Second, the FHWs in the study of Taal et al. [36] received a two-day training provided by two dermatologists, and trainees were afterwards assessed by a test. Only FHWs who received an 80% pass mark on their test were allowed to take part in the study. In the current study, the FHWs were purposely selected as FHWs with limited dermatological knowledge. They received a half-day training under guidance of NLR staff only to become familiar with the functionalities of the NLR SkinApp and on data collection.

The percent agreement among the FHWs was 58% in our study [44]. By contrast, the percent agreement among the dermatological experts/dermatologists, as might be expected, turned out to be much higher (96%); however, 21 patients were excluded because no final diagnosis could be made by the third dermatologist. Including these 21 patients would have led to a slightly lower percent agreement among the dermatological physicians. A matching dermatologist diagnosis was chosen as reference standard in this study, as further testing such as pathological examination using skin biopsies—often regarded as the gold standard in dermatology—is commonly unavailable in areas targeted by the NLR SkinApp and included in this study. In addition, skin biopsies are often not required according to medical guidelines, and neither desired for ethical/patient-friendly reasons, for many of the common skin diseases found in this study (e.g., tinea capitis, atopic dermatitis).

There is still much to gain in improving the functionalities and the validity of the NLR SkinApp in support of the performance level of the FHWs. The validity of the NLR SkinApp could be increased by specifying the content of the application per country taking into account the different dermatological epidemiology, by improving the decision tree, by giving more weight to certain symptoms and signs in relation to certain diseases, and by adding a few diseases such as urticaria, keloid, and folliculitis because they were commonly diagnosed in this study as part of the ‘other’ category in the NLR SkinApp. Further studies aimed at improving the content of the NLR SkinApp are advised. To increase the performance level of the FHWs, the number of training days may have to be extended. In the general medical curriculum of FHWs in these settings, dermatological training is often limited, for example, counting only 0.5–1 day in their entire curriculum (field reports) [14]. Furthermore, a teledermatology component to enable FHWs to consult a specialist when needed might be of added value.

The most frequently diagnosed diseases in this study were similar to findings in other studies: eczema, acne, pityriasis versicolor, tinea capitis, scabies, and vitiligo [1,5,13,17,18]. With 15.2% and 23.8%, respectively, eczema was the most common seen skin condition in the research areas in Tanzania and Ethiopia. This is similar to prevalence studies conducted in these countries, with frequencies ranging from 18.5 to 43.7% [13,17,18]. In contrast, this study found a lower number of tinea capitis cases: 3.6% compared to 11–59.2% in prevalence studies [13,28,36]. However, tinea capitis is most likely to develop in prepubertal children [45,46]. The number of patients in this study aged 14 years or below (13.3%) was significantly lower compared to other studies (51.2–74.8%), which may explain the lower number of tinea capitis cases we found [13,28,36]. In the research areas in Tanzania, other commonly seen skin conditions were acne (6.3%) and pityriasis versicolor (5.2%). In comparison with studies in the same country, higher prevalence rates for acne (19.2%) were seen in the study of Satimia et al. [13], but lower prevalence rates for pityriasis versicolor (0.7%) were found in the study of Mponda et al. [17]. Vitiligo and angular cheilitis were commonly seen skin conditions in the research areas in Ethiopia. Similar prevalence rates for vitiligo (8.9%) and angular cheilitis (7.6%) were found compared to another study conducted in Ethiopia [47]. Taken together, differences were found in the number of patients per skin disease between the two countries and within the available literature. This may be a result of sociogeographic factors, as well as the limited number of patients included in this study.

Although this study purposely selected FHWs with limited dermatological knowledge, their knowledge was not assessed as part of the selection process, and this study does not show the level of knowledge of the FHWs in case they are not using the NLR SkinApp. This makes it difficult to determine the extent of the contribution or added value that the application makes to the FHWs’ capacity to diagnose and treat skin diseases. In a following study, it would be interesting to measure the difference in diagnosing skin diseases with and without the use of the NLR SkinApp by the FHWs in order to examine the diagnostic contribution of the NLR SkinApp. Moreover, this study included two FHWs per country. In a further study, it may be valuable to include a wide variety of FHWs.

After this study, the NLR SkinApp has been integrated into the WHO SkinNTD app which was launched in October 2023. This has led to an enriched application to strengthen health workers’ capacity to serve patients with skin diseases [48]. Recommendations from this study were taken into account, and the diseases folliculitis, keloid scar, and urticaria, as well as noma (cancrum oris or gangrenous stomatitis) were added in both the NLR SkinApp and WHO Skin NTD App [49,50]. Noma was chosen as additional disease to be included in the app because of the rapid progression of this condition with high risk on mortality/disability and existing lack of awareness by healthcare workers [51]. In December 2023, WHO officially recognizes noma as a NTD, bringing the total number of NTDs to 21 [52]. The quality of the version of the integrated WHO SkinNTD app was further increased by adding advanced options such as country specific settings and artificial intelligence. Additional studies on the usability of this new app are expected soon.

## 5. Conclusions

The FHWs using the NLR SkinApp diagnosed 45% of the skin disease accurately. Sensitivities ranged from 23.0% to 76.9%. Management recommendations given had potential negative effects or potential severe effects in 37.0% of the management recommendations given by the FHWs. As stated, this study did not look at FHWs not using the NLR SkinApp. Inter-rater reliability among the FHWs was low, which indicates that there is potential to improve the diagnostic support of the NLR SkinApp. Further studies with a bigger sample size are needed to investigate the added value of the NLR SkinApp in supporting FHWs to diagnose and treat skin diseases, highlighting that the application is a supportive tool for FHWs to diagnose, and not a diagnostic tool. The results for eczema, for which the sample size was sufficient, are encouraging and demonstrate the potential of the NLR SkinApp. Improving the decision tree embedded in the NLR SkinApp and adding skin diseases which were commonly seen during this study under the category ‘other’, including urticaria, keloid, folliculitis, lichen planus, and tinea pedis, were identified as next steps in the development of the application. These steps have been followed-up in the integrated version, the WHO SkinNTD app which can be downloaded from Google Play Store and the AppStore (see supplemental materials for links).

## Figures and Tables

**Table 1:** Skin diseases included in the SkinApp.

Common Skin Diseases	Skin-Related Neglected Tropical Diseases (NTDs)	Other Included Skin Diseases
Acne Atopic eczema Impetigo Pityriasis versicolor * Psoriasis Scabies * Seborrheic dermatitis * Angular cheilitis * Herpes simplex * Herpes zoster * Molluscum contagiosum * Oral candidiasis * Pruritic popular eruption * Tinea capitis * Tinea corporis *	Buruli ulcer Cutaneous leishmaniasis Creeping eruption Leprosy Lymphatic filariasis Mycetoma Onchocerciasis Scabies * Yaws	Albinism Blistering diseases Kaposi Sarcoma * Vitiligo

* HIV/AIDS-related diseases.

**Table 2:** Definitions categories management recommendation.

Category	Definition
1—Correct	Correct diagnosis and management/Correct diagnosis but management not specified/(Partly) correct diagnosis and management
2—Neutral	Incorrect management but no potential negative effects/Incorrect diagnosis, condition not treated but no potential negative effects, or referred/Incorrect diagnosis but beneficial management, partly incorrect or partly correct diagnosis
3—Potential negative effects	Incorrect management, potential negative effects/Condition not treated, potential negative effects if not referred and/or treated appropriately or incomplete/Incorrect diagnosis, but (partly) beneficial management, referral needed
4—Potential severe effects	Incorrect management potentially leading to permanent damage or permanent morbidity if not referred and/or treated appropriately/Incorrect or no management, potential severe effects, or life threatening if not referred and/or treated appropriately

**Table 3:** Overview of the 10 most common skin diseases in the category ‘other’.

Condition	Number of Cases	Percentage
Urticaria	32	15.8
Keloid	14	6.9
Folliculitis	13	6.4
Lichen planus	12	5.9
Tinea pedis	11	5.5
Lupus erythematosus	10	5.0
Photodermatitis	10	5.0
Lichen simplex	8	4.0
Onychomycosis	7	3.5
Prurigo nodularis	6	3.0

**Table 4:** Diagnostic accuracy of five (categories of) skin diseases.

Skin Condition/Category	Se% (95% CI)	Sp% (95% CI)	PPV% (95% CI)	NPV% (95% CI)
Eczema	76.9 (70.6–82.4)	69.6 (66.0–73.0)	44.3 (41.0–47.7)	90.5 (88.2–92.5)
Tinea capitis/corporis	26.8 (15.8–40.3)	95.7 (94.1–96.9)	29.4 (19.6–41.7)	95.1 (94.3–95.8)
Skin-related NTDs	37.5 (24.9–51.5)	92.2 (90.1–93.9)	24.4 (17.6–32.8)	95.6 (94.7–96.4)
HIV/AIDS-related skin diseases	23.0 (16.5–30.6)	86.9 (84.2–89.2)	26.0 (19.8–33.2)	84.9 (83.7–86.1)
Acne	32.1 (20.3–46.0)	99.3 (98.4–99.7)	75.0 (55.4–87.9)	95.6 (94.8–96.3)

Se = sensitivity; CI = confidence interval; Sp = specificity; PPV = positive predictive value; NPV = negative predictive value.

## Data Availability

The datasets generated and/or analysed during the current study are available in the [NAME] repository, https://www.infontd.org/sites/default/files/2023-08/220222%20Dataset%20SkinApp%20validation_InfoNTD.pdf.

## Supplementary Materials

Link to the NLR SkinApp in AppStore and Google Play Store and WHO Skin NTD App. https://play.google.com/store/apps/details?id=com.appelit.leprastichting, https://apps.apple.com/nl/app/skinapp-nlr/id1228305837.

## List of Abbreviations

AAERC	AHRI/ALERT Ethical Review Committee
AI	Artificial intelligence
App	Application
CREC	CUHAS/BMC Research and Ethical Committee
FHWs	Frontline health workers
LF	Lymphatic filariasis
mHealth	Mobile Health
NGO	Nongovernmental organization
NTDs	Neglected tropical diseases
WHO	World Health Organization
